# Rapid culture-based detection of living mycobacteria using microchannel electrical impedance spectroscopy (m-EIS)

**DOI:** 10.1186/s40659-017-0126-7

**Published:** 2017-06-10

**Authors:** Roli Kargupta, Sachidevi Puttaswamy, Aiden J. Lee, Timothy E. Butler, Zhongyu Li, Sounak Chakraborty, Shramik Sengupta

**Affiliations:** 1 Department of Bioengineering, University of Missouri, 252 Agricultural Engineering Building, 1406 E. Rollins Street, Columbia, MO 65211 USA; 2 Department of Statistics, University of Missouri, Columbia, MO USA

**Keywords:** Mycobacteria, Tuberculosis, Electrical impedance spectroscopy, BD Bactec MGIT 960, *M. bovis BCG*, *M. smegmatis*

## Abstract

**Background:**

Multiple techniques exist for detecting *Mycobacteria*, each having its own advantages and drawbacks. Among them, automated culture-based systems like the BACTEC-MGIT™ are popular because they are inexpensive, reliable and highly accurate. However, they have a relatively long “time-to-detection” (TTD). Hence, a method that retains the reliability and low-cost of the MGIT system, while reducing TTD would be highly desirable.

**Methods:**

Living bacterial cells possess a membrane potential, on account of which they store charge when subjected to an AC-field. This charge storage (bulk capacitance) can be estimated using impedance measurements at multiple frequencies. An increase in the number of living cells during culture is reflected in an increase in bulk capacitance, and this forms the basis of our detection. *M. bovis BCG* and *M. smegmatis* suspensions with differing initial loads are cultured in MGIT media supplemented with OADC and Middlebrook 7H9 media respectively, electrical “scans” taken at regular intervals and the bulk capacitance estimated from the scans. Bulk capacitance estimates at later time-points are statistically compared to the suspension’s baseline value. A statistically significant increase is assumed to indicate the presence of proliferating mycobacteria.

**Results:**

Our TTDs were 60 and 36 h for *M. bovis BCG* and 20 and 9 h for *M. smegmatis* with initial loads of 1000 CFU/ml and 100,000 CFU/ml respectively. The corresponding TTDs for the commercial BACTEC MGIT 960 system were 131 and 84.6 h for *M. bovis BCG* and 41.7 and 12 h for *M smegmatis*, respectively.

**Conclusion:**

Our culture-based detection method using multi-frequency impedance measurements is capable of detecting mycobacteria faster than current commercial systems.

## Background

Tuberculosis (TB) is a chronic disease with a high mortality rate. According to a recent WHO report, it is estimated that about 1.5 million people died from TB in 2013 [1]. However, proper and timely diagnosis and treatment can reduce the mortality rate and the economic burden associated with TB [1, 2]. An early diagnosis of TB would yield two benefits. Firstly, clinical intervention (treatment with first-line antibiotics) is initiated sooner and secondly, drug susceptibility testing (DST) can be completed earlier, leading to tailored treatment of patients according to the DST results [3] and prevent the widespread transmission of drug resistant strains.

Various diagnostic techniques are available to clinicians to identify patients with TB and/or detect mycobacteria in patient samples. Common diagnostic techniques include serial sputum smear microscopy and chest X-ray. However, these techniques suffer from poor specificity and/or sensitivity (have high rates of false negatives) and information on drug susceptibility is lacking [1, 3–5]. An alternative testing method is the Mantoux tuberculin skin test (TST). In Mantoux TST, the response to the injection of a small dose containing *M. tuberculosis* derivatives is observed at different time intervals. However, this test suffers from low specificity and the interpretation of the obtained test results is subjective [2]. Other techniques include polymerase chain reaction (PCR) based rapid diagnostic tests [6–8]. The Xpert MTB/RIF is a nucleic acid amplification technique that can detect the presence of mycobacteria in less than 2 h, and has a limit of detection of 131 CFU/ml in sputum [9]. However, it suffers from a couple of limitations when compared to automated culture based systems. Firstly, the limit of detection of culture-based systems is 1 CFU in the sample [10, 11]. Second, the reagents/chemicals used are sometimes unstable in “real-world” situations [4], which is not the case for disposables of the culture-based systems (growth media). Finally, whereas a hardware unit for culture-based system (BD Bactec 9120 that handles 120 Myco/F-lytic bottles simultaneously) costs ~$20,000 and disposables cost <$10 per test, the costs are significantly higher for the Xpert [12]. The XpertMTB/RIF instrument (with 4 modules that can run 16–20 tests per 8 h shift) has an unsubsidized cost of ~$34,000 and each single use cartridge has a cost of ~$40 [13]. The high cost is especially daunting to end-users (hospitals) in low to mid-resource environments, where the great majority of TB cases occur. While, a consortium of charitable organizations that include the Foundation for Innovative New Diagnostics (FIND), the Gates Foundation, USAID and UN agencies, provide a 50% subsidy on the instrument and a 75% subsidy on the disposables, making the Xpert™ instrument available for $17,000 and disposables available for ~$10 a test, such funding is limited and available to only a few select “approved” users. Hence, culture based testing is still considered the gold standard [1] because of its limit of detection of 1 CFU and still widely used [14] due to their relatively low cost.

Culture based detection is cheap, reliable, highly accurate and can be used for DST against multiple drugs and drug combinations [1]. It has been found that in comparison to solid media, mycobacteria grow much faster in liquid media [15]. Currently a number of culture-based methods for detection of TB is available such as broth micro-titer based microscopic observation drug susceptibility (MODS) assay and automated liquid culture based diagnostic systems such as the BD BACTEC 960 MGIT fluorometric system, BACTEC 460 TB System and Biomerieux’s BacT/ALERT 3D system for detecting mycobacteria in samples. MODS assay, though inexpensive and has an average time-to-detection (TTD) of ~8 days [16], requires training and technical expertise of the personnel [15]. On the other hand, automated liquid culture based systems only need sample to be prepared and loaded into the instrument. A major drawback of these broth based culture systems (automated, or otherwise) is that they are time consuming, with most positive clinical samples having TTDs of ~14 days (for MGIT system) [17]. Also samples are typically incubated for at least 6 weeks before being deemed negative [18, 19]. Hence, a system for detecting mycobacteria that retains all the advantages of current automated culture based systems like the MGIT system while requiring less time for results, is likely to be welcomed by users.

In 2010, Association of Public Health Laboratories (APHL) and the US Centers for Disease Control and Prevention (CDC) launched a 118-questions survey to assess the capabilities and capacities of TB testing facilities in United States [14]. Of the 656 respondents, 580 (~88%) of them had know-how for implementing TB testing. Out of them, 466 facilities used primary broth based culture and 356 (~76%) of them used semi/fully automated culture base systems. (The University of Missouri Hospital also uses a fully automated culture system viz. the BACTEC™ MGIT ™ 960 System from BD).

TB detection in bacterial suspensions by automated broth based culture system takes a long time, chiefly because of the mechanism by which these systems operate. The semi-automated BACTEC 460 TB system uses Middlebrook 7H12 broth medium containing radioactive ^14^C radioisotopes, and uses radiometric detection of ^14^CO_2_ released to detect the presence of active microorganisms (mycobacteria) [20, 21]. MGIT 960 systems, on the other hand, contain an oxygen-quenched fluorochrome that changes color as oxygen levels in the media change. MGIT 960 systems, hence, detect the depletion of oxygen inside the tubes as living mycobacteria consume oxygen [22]. Similarly, systems like Versa TREK and MB/BacT-Alert are based on monitoring headspace pressure changes of sealed bottles/tubes [21] and monitoring the pH change of the media, respectively [23]. Thus, all these automated culture based systems share a basic operating principle viz. they sense changes in medium properties (O_2_/CO_2_ levels, pH etc.) brought about by mycobacterial metabolism as a marker for actively respiring/proliferating microorganisms. In this respect, they are similar to automated systems used for other applications such as blood culture (BACTEC™ and BacT/Alert™) or food quality testing (RABIT, Malthus 2000 etc.) [24].

The term “Impedance Microbiology” is often used to describe techniques that are based on the ability of microorganisms to alter the electrical properties of their growth media. The fact that microbial metabolism causes an increase the conductivity of the medium by breaking down less conductive species like sugars and proteins into more conductive ones such as lactic/pyruvic/carbonic acids and urea/ammonia was first reported by Stewart in 1899 [25], and was studied in a quantitative manner by Cole [26].

**Fig. 1:**
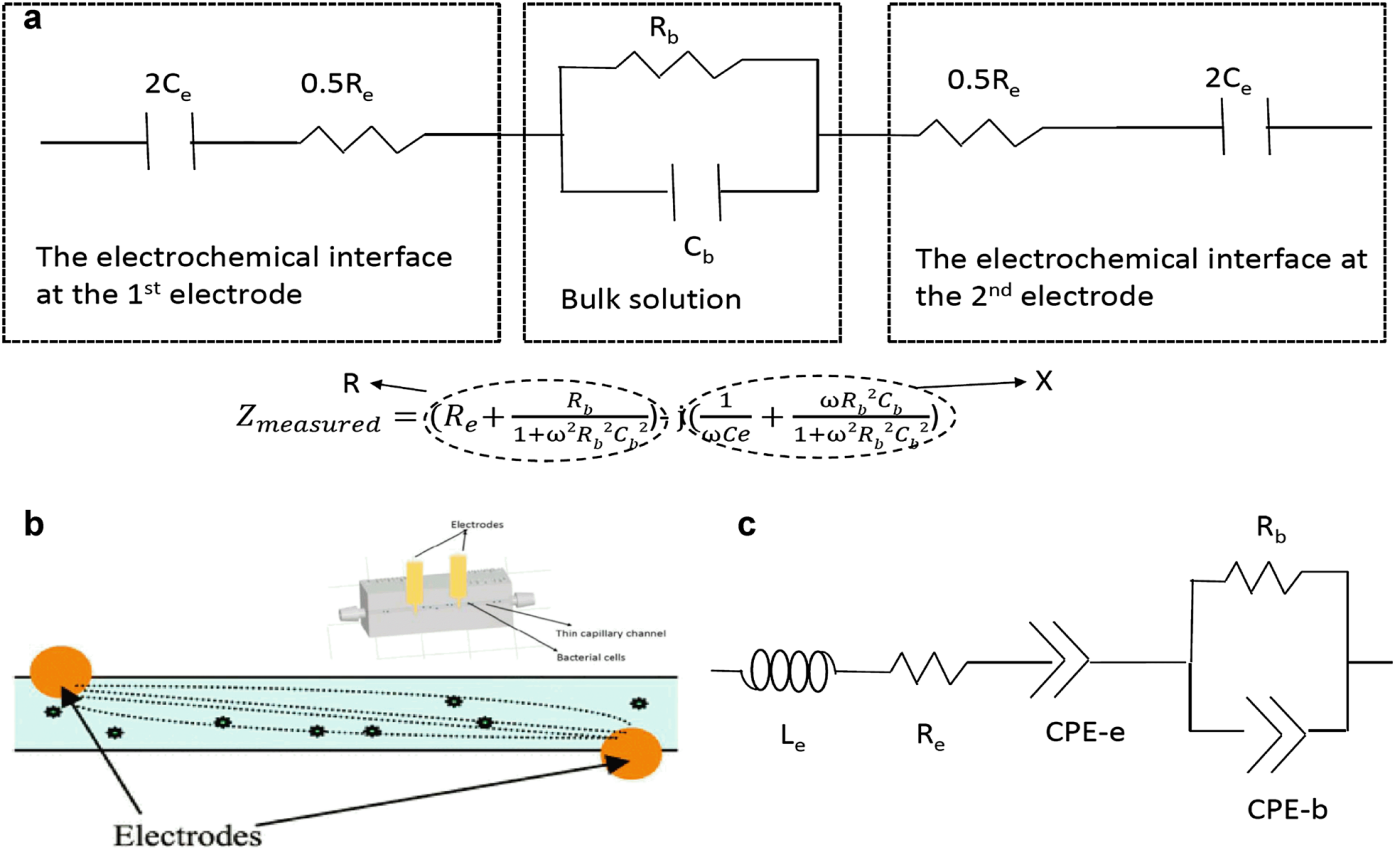
Electrical model and cassettes used for impedance measurement. **a** Electrical model representing two electrodes submerged in a microorganism suspension enclosed in a thin capillary channel. **b** 3D printed long and narrow microfluidic channels with two gold electrode inserted for impedance measurement (*inset*) and schematic showing electrical lines of forces between two electrodes in the channel [24]. **c** Modified electrical model which uses CPE instead of ideal capacitors to detect the presence of microorganisms in a suspension

After the development of more accurate and robust instruments to measure and monitor impedance, this approach became more feasible to implement in a lab setting. A number of researchers began investigating this method more rigorously and started to explore the use of this technique as a means to detect the presence of bacteria in various settings. In particular, it was discovered [27] that two distinct sources contribute to any measured electrical signal (impedance): the bulk solution and the electrochemical interface of the solution and the electrodes in direct contact with it. The bulk solution typically contains two types of species: (a) relatively mobile ions (such as Na+, K+, Cl−, PO_4_—etc.) that move through the solution overcoming drag by the solvent molecules on the application of an electric field, and (b) larger, relatively immobile species such as proteins and cells that either carry native charge or on which charges could be induced on applying an electric field. Thus, as shown in Fig. 1a, the bulk could be represented by a resistor and capacitor in parallel, and the electrochemical interface of the electrode and solution, which consists largely of ions in chemical equilibrium with the bulk, could be represented by a capacitor in series with the resistance of the electrode itself [24, 27]. By increasing the number of ionic species present, bacterial metabolism is found to affect not only the bulk resistance [28], but also the surface capacitance [29, 30] since the latter arises from ions in chemical equilibrium with those present in the bulk. A more rigorous analysis of the relative quantitative contributions of the bulk and the interface to the overall capacitance [31] found that at operating frequencies of 1 MHz and lower, the contributions from the bulk capacitance (a.k.a. the geometric capacitance) and the interfacial capacitance were of the order of picofarads (10^−12^ F) and micro to nano farads (10^−6^ to 10^−9^ F), respectively. Thus, while it is known that exposure to an AC field causes charge accumulation at the membrane of cells with non-zero membrane potential (living cells) [32], it logically follows that an increase in the number of cells in suspension would result in increased charge storage (bulk capacitance). Measuring changes in bulk capacitance brought about by an increase in the number of suspended cells, however, was not considered feasible. The consensus was explicitly stated in a review article by Munoz-Berbel et al. [33], who wrote that

*“Geometric capacitance… due to the solution between the electrodes …. Because of its small value, in the picofarad range, it can usually be neglected in the measurement frequencies used in biosensor applications”*

Our early work [24] at attempting to measure changes in bulk capacitance (C_b_) relied on using geometric effects to enhance the effect of changes in C_b_ to the measured reactance (X) (the “imaginary” or “out-of-phase” component of the impedance). As shown in Fig. 1b, the use of long narrow microfluidic channel causes a larger fraction of the electrical flux lines to interact with the (few) microorganisms present. Another way to look at the effect is to study the equation embedded in Fig. 1a. Since for any given material, the resistance is inversely proportional to cross-sectional area and directly proportional to length, the long narrow geometry results in an increase in bulk resistance (R_b_). It can be seen that for the reactance (X), the C_b_ is always multiplied by R_b_. Thus, any changes to the value of X due to a change in C_b_ will be “magnified” by the higher R_b_. Since the R_b_C_b_ is also multiplied by the frequency (ω), this effect is further enhanced at high frequencies. The geometric effect alone allowed us to detect changes in C_b_ that was previously considered too small to measure.

We further enhanced the sensitivity of our measurement technique by using AC signal with higher frequencies (ω) as high as 100 MHz. At these frequencies, the charge on the electrode reverses every ~10 ns (as opposed to 1 µs or longer for signals of frequency 1 MHz or lower). A consequence of this is that, certain assumptions that we had previously made regarding the electrical behavior of the solution, no longer hold. For instance, the model shown in Fig. 1a assumes that the capacitances (bulk and interfacial) were ideal. In an ideal capacitor, charges accumulate instantaneously. However, in cases like ours charge carriers are ions that are slow and bulky compared to electrons, and may take as long as hundreds of nanoseconds to complete the process of accumulation at the electrode [34]. Hence, when fitting data, we replace the ideal capacitors in equation in Fig. 1a with constant phase elements (CPEs) (as shown in Fig. 1c). The impedance of a CPE is mathematically given [35] by

1 $${\text{Z}}_{{{\text{CPE}}}} =0+{\text{1}}/\left({{{\text{j}\upomega }}}\right)^{{\text{n}}}{\text{Q}},$$

as opposed to an ideal capacitor, whose impedance is given by

2 $${\text{Z}}_{{{\text{capacitor}}}}=0+{\text{1}}/{\text{j}{\upomega\text{C}}}$$

Q is the magnitude of the CPE and n is its phase. It can be seen that when n equals 1, the two equations are identical. In addition, at the high frequencies we deal with (up to 100 MHz), the inductance of the wires connecting to the test circuit (the electrodes in contact with the aqueous suspension) can no longer be neglected. It is given by

3 $${\text{Z}}=0+{\text{j}{\upomega\text{L}}},$$

where L is the inductance and ω is the angular frequency of the applied voltage. While this results in a more complicated circuit model with 7 parameters (the resistances of the electrode and solution, the magnitudes and phases of the CPEs at the electrode and in the bulk, and the connection inductance), we use a large number of data points (Z at 200 different frequencies) and commercially available software for electrochemical analysis (Z-view™) to obtain the values of our parameters.

By being able to detect small changes in C_b_, our measurement method can tell us when the bacterial numbers in suspension are increasing, even when their values are extremely low (~1000 CFU/ml). It can hence serve as a faster alternative to traditional automated culture based detection methods like those discussed earlier. We have earlier demonstrated that our method is able to achieve faster times to detection (TTD) in suspension with low initial loads (<100 CFU/ml) for applications such as food quality testing [36] and blood culture [37]. In both cases, we obtained 4- to 10-fold reductions in TTD, when compared to commercially available culture based systems like BACTEC etc. [37]. In this work, we seek to demonstrate (a) a similar approach can also be used to detect/rule out the presence of mycobacteria in broth based culture systems, and (b) that systems using our approach would have a lower TTD than state of the art culture-based detection systems like MGIT 960 system, Versa TREK and MB/BacT-Alert. We believe that shorter TTDs may turn out to have clinical relevance in the early diagnosis (and hence treatment) of persons with TB.

## Methods

### Background and overview

*Mycobacterium bovis* BCG and *Mycobacterium smegmatis* is used instead of *Mycobacterium tuberculosis* (Mtb) to demonstrate the applicability of our method in vitro since they are easier and safer to handle under BSL-2 laboratory condition [38, 39]. *M. bovis* BCG, like Mtb, is a slow growing mycobacterium with a doubling time of ~20 h [40]. *M. smegmatis,* on the other hand, is a rapidly growing mycobacterial species having a doubling time of ~3 h, but has membrane properties similar to Mtb [41, 42].

To ensure absence of other bacterial contaminants all suspensions are frequently stained by Kinyoun staining. To visualize mycobacteria, acid-fast staining is used as the presence of mycolic acid in the cell walls prevent other staining protocols from working [43, 44].

**Fig. 2:**
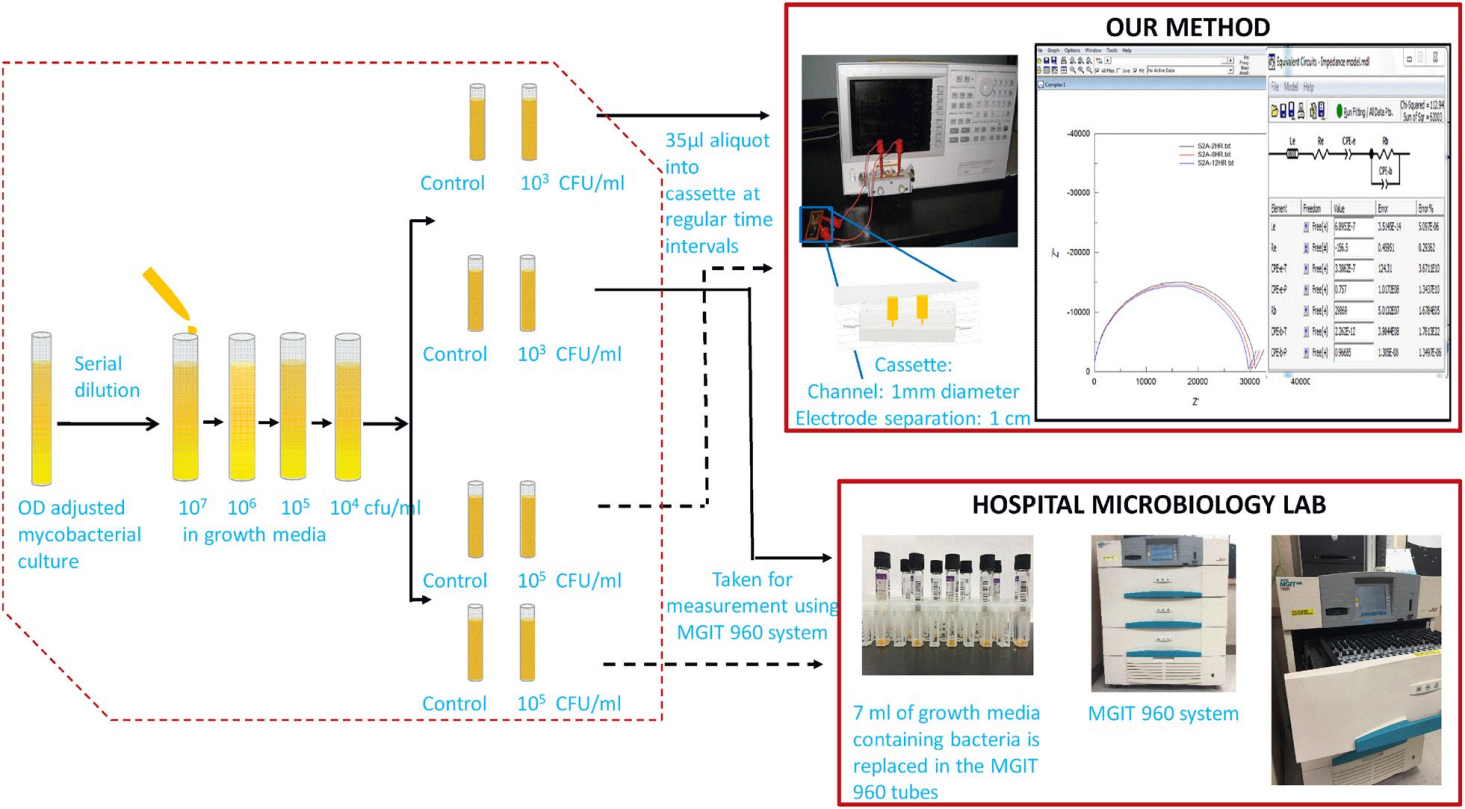
Experimental set-up for determining TTD of mycobacteria. Two sets of similar samples are prepared by inoculating with mycobacteria and incubated at 37 °C. One set is sent to the hospital microbiology lab for detection using MGIT 960 automated culture-based system while the other set of sample is tested using our technique. For our technique, at regular intervals of time, small aliquots of sample are drawn and its impedance is tested using impedance analyzer. The data is fitted to an equivalent circuit to generate a Nyquist plot [48] and thereby estimates the values of the desired circuit parameters. We show on the *graph* three sets of data: those obtained at 2, 8, and 12 h for a culture of *M. smegmatis* (*large*, *medium*, and *small semi-circles*). The *inset* shows the software output for circuit parameters obtained by fitting the data at 12 h to the circuit

Figure 2 shows the broad outline of the experimental protocol. Individual steps/procedures are described in detail later. Briefly, active cultures of *M. bovis* BCG and *M. smegmatis* are created in BD MGIT media and Middlebrook 7H9 media respectively with initial loads of 1 × 10^3^ and 1 × 10^5^ CFU/ml. One set of cultures is sent to an external lab for analysis using an automated system (BD BACTEC MGIT 960). The automated system records when individual culture-tubes are placed into it, and when growing mycobacteria are detected by it for each individual tube, thus allowing us to easily obtain the time-to-detection (TTD). The other set is analyzed in our lab using our method.

In our method, tubes containing the cultures are incubated at 37 °C. Periodically, aliquots from these growing cultures are withdrawn and placed within a 3D printed microfluidic cassette with 1 mm diameter micro-channels (Fig. 1b). Microchannel electrical impedance spectroscopy (m-EIS) data is obtained from the cassette and analyzed to obtain the bulk capacitance (C_b_). Multiple sampling is done to ensure repeatability. C_b_ values are compared with the baseline (initial) values, and the time taken for the measured values to become larger than the baseline (with statistical significance) is our TTD.

Control samples containing growth media but no microorganisms are also analyzed similarly. TTDs of similarly prepared samples (containing approximately the same initial load of microorganisms) for the two methods (ours and that of the MGIT system) are compared.

### Mycobacterial cell culture

Slow growing acid-fast organism *M. bovis BCG* (ATCC^®^ 35734™) and rapid growing mycobacteria *M. smegmatis* (ATCC 700084) are used. Colonies of these mycobacteria are obtained by plating on egg based Löwenstein-Jensen plates for *M. bovis BCG* and 7H10 agar plates for *M. smegmatis*. Suspension cultures of the same are obtained by inoculating BD MGIT media supplemented with mycobacteria growth supplements in the case of *M. bovis BCG*, and Middlebrook 7H9 broth supplemented with 0.05% Tween 80 and Middlebrook Albumin Dextrose Catalase supplements (ADC) in the case of *M. smegmatis*. During sub-culturing, the bacterial suspension is incubated at 37 °C with continuous agitation. The optical density (OD) mid-log cultures of the microorganisms are then adjusted to OD_600_ = 0.05 for *M.bovis* BCG and OD_600_ = 0.1 for *M. smegmatis* using a spectrophotometer which corresponds to a value of 5 × 10^6^ CFU/ml and 1–5 × 10^7^ CFU/ml respectively for use in the study [45–47].

### Microchannel electrical impedance spectroscopy (m-EIS)

The basic principles governing the use of m-EIS to detect microorganisms have been described in our prior work [36, 37, 48]. Briefly, the protocol requires us to periodically (every 12–24 h for *M. bovis BCG*, and 2–4 h for *M. smegmatis*) perform an electrical “scan” of sample aliquots in a microfluidic cassette, wherein we measure electrical impedance at multiple (200) frequencies ranging from 1 kHz to 100 MHz. The cassette contains a 1 mm diameter microchannel with two gold electrodes, 1 cm apart in the channel. An AC voltage of 500 mV is applied across the two gold electrodes, using an Agilent 4294A Impedance Analyzer. At each frequency (ω), both the in-phase and out-of-phase components of the electrical impedance, Z, [resistance (R) and reactance (X)] are measured. In order to take the EIS measurements (scans), all aliquots from a given culture (across the different points in time) are introduced into the same individual cassette. As the cassettes used are handmade their readings vary from each other slightly and hence the data (values of bulk capacitance obtained) is scaled with respect to the value at the initial point in time (on the same cassette) to account for the cassette to cassette variation.

The Z vs. ω data is fitted to an equivalent electrical circuit shown in Fig. 2 using a commercially available software package (Z-view™). The software provides an estimate for the various circuit parameters, including the “bulk capacitance”, which happens to be our parameter of interest—that provides a measure of charges stored in the interior of the suspension (away from the electrodes). It may be noted that the bulk capacitance is represented as a constant-phase element (CPE) to account for the non-ideal nature of the capacitance at cell membranes. The magnitude of the CPE, thus, reflects the amount of charge stored at the membranes of living microorganisms in suspension. Any increase in the number of microorganisms in suspension should hence, in theory, lead to larger amounts of charged stored in the interior of suspensions, and hence lead to a higher bulk capacitance (CPE_b_-T) over time.

When observing a given suspension suspected of harboring proliferating microorganism, our problem reduces to asking the question of “Is the current value of the bulk capacitance *significantly* greater than its value at the initial point in time?” To enable us to answer this question with a greater degree of confidence, for each sample, capacitance of 4–5 replicates are measured at specified time interval and statistically compared to baseline using Mann–Whitney U test. The earliest time point at which a significant difference is found, is defined as the TTD by m-EIS. Details of the statistical method are provided below.

### Statistical analysis

Statistical analysis is performed in Microsoft Excel using Mann–Whitney U test. This non-parametric test compares if the population average between two groups is significantly different or not [49]. We chose to adopt the Mann–Whitney U test over the more popular tools like *t* test since we have only a few data points (bulk capacitance readings) per time point (5 for *M. bovis* BCG cultures, 4 for *M. smegmatis* cultures). More importantly, the normality assumption of the reading which is required for a t test is not appropriate for our data. To check if the average of the bulk capacitance obtained at a time interval is significantly different from the bulk capacitance reading obtained in the first reading, the mean of the readings taken at the latter point in time is compared with the mean of the readings at the beginning of the culture (baseline values) and the U values corresponding to a p value of 0.05 (level of significance of 5%; two tailed test) are calculated. Our null hypothesis is that the two bulk capacitance values are equal and the alternate hypothesis is that there is a significant difference between the bulk capacitance values. The Mann–Whitney U value obtained for our readings is compared to the critical U value (2 in the case of *M. bovis* BCG cultures where we had 5 readings at each time point and 0 in the case of *M. smegmatis* cultures where we had 4 readings at each time point) [49]. If the Mann–Whitney U value obtained is equal to or less than the critical value, the null hypothesis is rejected, which means that there is a significant difference between the bulk capacitance values at the two time points. The earliest point in time where the U values obtained are equal to, or lower than, the critical U value is our time-to-detection (TTD) for a given sample.

## Results

Six different kinds of suspensions are studied: Two of them contained *M. bovis* BCG in MGIT media with initial loads of ~10^3^ CFU/ml and ~10^5^ CFU/ml, respectively. Two other suspensions contained *M. smegmatis* in 7H9 media, again with initial loads of ~10^3^ CFU/ml and ~10^5^ CFU/ml. The other solutions are controls, consisting of sterile MGIT and 7H9 media. One sample each of the controls, three samples each of the suspensions with *M. bovis BCG*, and two samples each of the suspensions with *M. smegmatis* are analyzed using our m-EIS method. In parallel, one sample of each of the suspensions is sent to the University of Missouri Hospital Microbiology lab for analysis using the BD BACTEC MGIT™ system. Times-to-detection (TTDs) for our method are compared to those of the MGIT system.

**Fig. 3:**
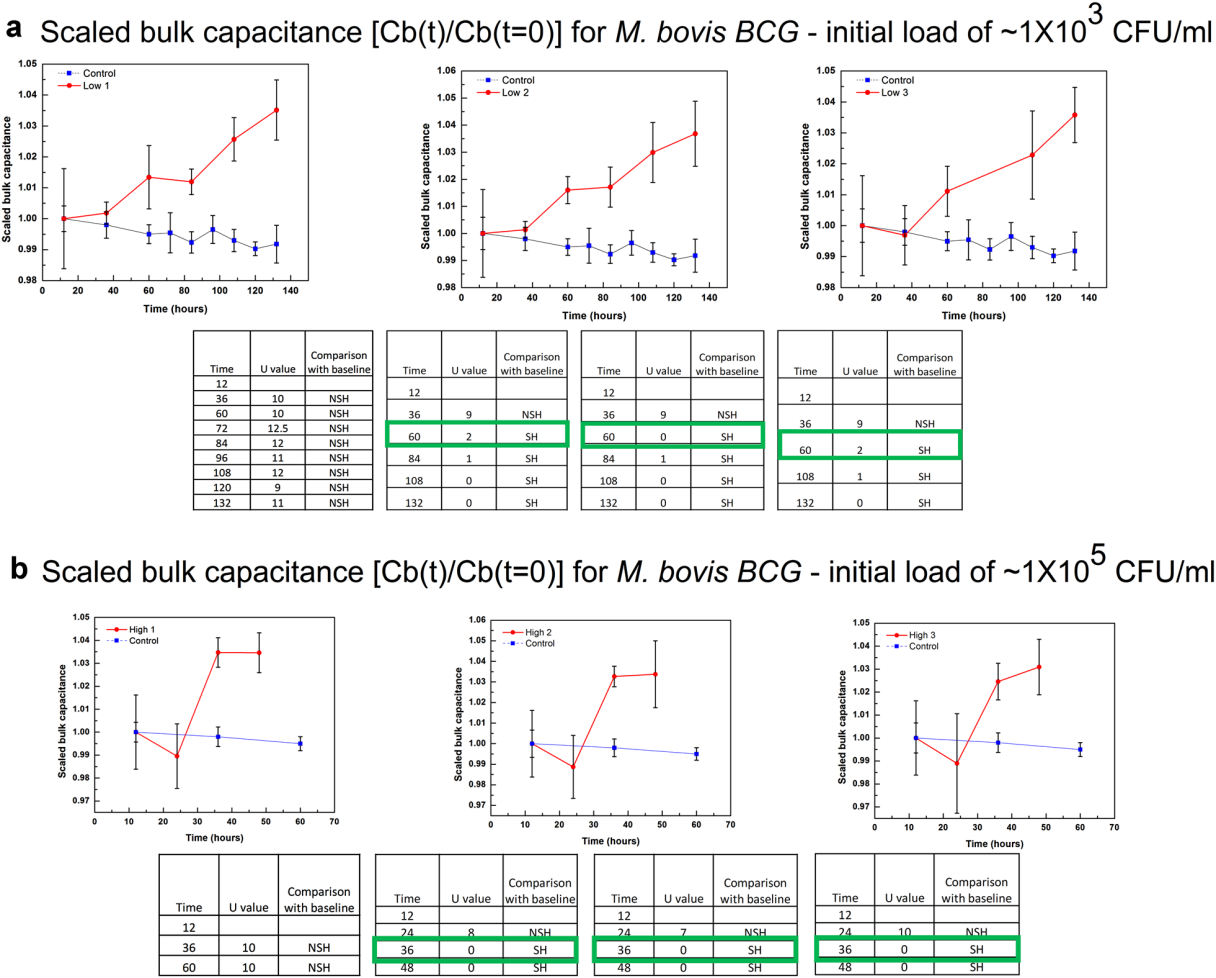
TTD of *M. bovis BCG* samples. Plot of bulk capacitance obtained over time for samples with **a** low initial loads (~1 × 10^3^ CFU/ml) and **b** high initial loads (~1 × 10^5^ CFU/ml) for *M. bovis BCG*. The *error bars* indicate the standard deviation of the readings (n = 5) taken at each time interval. Statistical analysis is used to compare the baseline reading with the various time interval readings (U_critical_ = 2). [*NSH* not significantly higher, *SH* significantly higher]

**Fig. 4:**
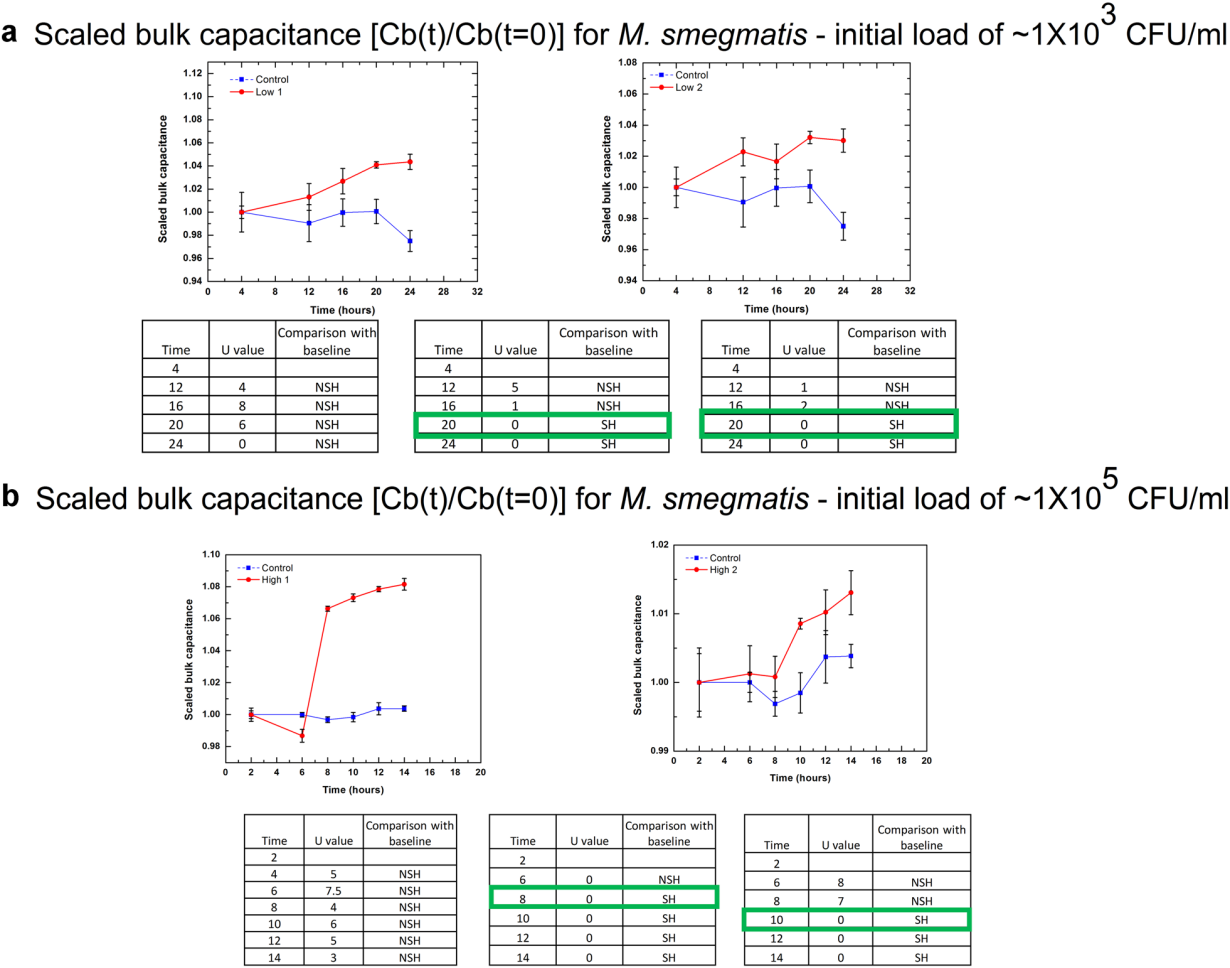
TTD of *M. smegmatis* samples. Plot of bulk capacitance obtained over time for samples with **a** low initial loads (~1 × 10^3^ CFU/ml) and **b** high initial loads (~1 × 10^5^ CFU/ml) for *M. smegmatis*. The *error bars* indicate the standard deviation of the readings (n = 4) taken at each time interval. Statistical analysis is used to compare the baseline reading with the various time interval readings (U_critical_ = 0). [*NSH* not significantly higher, *SH* significantly higher]

Bulk capacitance values obtained on studying cultures initially containing ~10^3^ and 10^5^ CFU/ml of *M. bovis* BCG are shown in Fig. 3a, b (along with the corresponding control). As shown in the table included as part of the figure, a statistically significant increase in the value of the bulk capacitance is seen at 60 h for 10^3^ CFU/ml and 36 h for 10^5^ CFU/ml in all three cases. Similar plots are shown for the suspensions with initial loads of ~10^3^ CFU/ml of *M. smegmatis* and ~10^5^ CFU/ml of *M. smegmatis* are shown in Fig. 4a, b respectively. The TTDs obtained for each type of suspension using our method, and the TTD for similar solutions using the commercially available system (MGIT) is shown in Table 1.

**Table 1:** Comparison of TTD values obtained by our technique to BD BACTEC MGIT 960 system

*M. bovis BCG*			*M. smegmatis*		
Bacterial concentration used (in CFU/ml)	Our technique TTD (in hours) (n = 3)	Hospital TTD (in hours) (n = 3)	Bacterial concentration used (in CFU/ml)	Our technique TTD (in hours) (n = 2)	Hospital TTD (in hours) (n = 2)
Control—no bacteria added	No growth	No growth	Control—no bacteria added	No growth	No growth
100,000	36 ± 0	84.6 ± 0.58	100,000	9 ± 1.41	12 ± 0.71
1000	60 ± 0	131 ± 11.79	1000	20 ± 0	41.7 ± 0.35

Compares the TTD values obtained for the various concentrations of *M. bovis BCG* and *M. smegmatis* by our technique and that obtained by the commercially available automated system BD BACTEC MGIT 960

## Discussion

For both mycobacterial species, and for both initial loads in each, our Times-to-Detection (TTDs) are lesser than the corresponding TTDs obtained by the hospital using a Bactec MGIT 960 system. Also, the lower the initial load, and the longer the organism’s doubling time, more is the time saved. It is thus anticipated that the difference in TTDs with our method and the commercially available culture based methods similar to those employed by the MGIT system, will be even greater for clinical samples containing *M. tuberculosis* (Mtb), since the doubling time of Mtb is even greater than that of *M. bovis BCG*, and the initial loads can be an order of magnitude lower than what we tested (~1000 CFU/ml). There are non-culture based systems like Xpert MTB/RIF which have a low limits of detection and can do rapid detection of mycobacteria (~2 h), these devices and reagents used are expensive. However, our technique, based on using the same reagents as that of the BACTEC MGIT 960 (and compatible with other reagents used for growing mycobacteria) will be cost-effective and will cut down the TTD considerably when compared to automated culture-based system.

We believe that our technique of detection, though faster, will have similar sensitivity and specificity as the existing culture based automated system. Human sputum from patients with TB contain not only mycobacteria, but also a large number of other gram-positive and gram-negative bacteria [50]. The current protocol for detecting mycobacteria using MGIT and similar automated culture based systems requires the user to first carry out a “decontamination” step that eliminates all the non-mycobacterial pathogens while preserving *M. tuberculosis*. Standard decontamination protocols exist, whose efficacy at killing non-mycobacterial pathogens while keeping *M. tuberculosis* cells viable and culturable has been extensively documented [51–54]. Nevertheless, it is not unheard of for non-mycobacterial species to survive decontamination, or for the process to kill or render unculturable some or all of the mycobacteria present [55, 56]. The former case leads to false positives, and the latter to false negatives in currently used automated culture-based systems like the MGIT. Another source of false positives is contamination during handling and processing, either with mycobacteria or with non-mycobacterial species [57]. On the other hand, false negatives can also arise due to inefficient sampling, wherein the sample of sputum used does not contain any *M. tuberculosis* cells (although the patient does have TB). This is more likely for patients with low mycobacterial loads [57]. When deployed for handling clinical samples, our system will also require the user to perform the same decontamination process that is used before placing the sample in other culture-based systems, and hence we will face the same potential pitfalls (mentioned above) that other systems face. Hence, we expect our diagnostic sensitivity and specificity to be similar to that of current systems like the MGIT system. Also, our limit of detection should remain 1 CFU, as with other culture-based systems.

For use in our system, we would require sample pre-treatment (decontamination) to be done using the same protocols that are currently used before samples are loaded into automated systems like MGIT. The sputum obtained from different people will be different and hence the initial C_b_ value will be different and will vary from person to person. However, in all cases, we will be looking for *significant increases* from the baseline value (whatever that value may be). A similar approach (looking for changes from the baseline-value) has been used successfully used to detect the presence of living bacteria in blood culture broth [37]. Like sputum, blood drawn from different people have different bulk capacitance values to start with. By looking for a significant increase in the C_b_ value from their respective initial values, we are able to account for sample-to-sample variations. Therefore, any change in the C_b_ value will be due to the change in the number of mycobacteria present in the sample.

An important limitation of the system as implemented in the work presented here is that aliquots are manually withdrawn at select points in time. With support from the NIH via a phase II SBIR award (No. R44-AI096572-02), our industry partners (Techshot Inc., and ImpeDx Diagnostics), are currently developing an automated blood culture system that includes an automated sampler. Such a system, which could be readily adapted for detecting mycobacteria in cultures as well, will be tested against standard of care equipment (Bactec and similar) in multiple locations.

## Conclusion

When trying to detect mycobacteria via culture, our times-to-detection (TTDs) are much lower than those obtained using currently available culture based detection systems like the MGIT™ by a factor of ~2. Since clinical samples take up to 4 weeks to yield a positive result, an automated system implementing our method (which we plan to develop and optimize) could potentially obtain results many days (and sometimes even 1–2 weeks) faster than currently available automated culture-based systems. This is likely to yield significant clinical benefits in terms of improved patient outcomes.

## Abbreviations

m-EIS: microchannel electrical impedance spectroscopy
TTD: time-to-detection
MGIT: mycobacteria growth indicator tube
ADC: bovine albumin, dextrose and catalase
OADC: oleic acid, bovine albumin, dextrose and catalase
CFU: colony forming units
WHO: World Health Organization
DST: drug susceptibility testing
TST: tuberculin skin testing
PCR: polymerase chain reaction
MODS: microscopic observation drug susceptibility
APHL: Association of Public Health Laboratories
CDC: Centers for Disease Control
C_e_: interfacial capacitance
C_b_: bulk capacitance
R_b_: bulk resistance
Mtb: mycobacterium tuberculosis
OD: optical density
ω: frequency
Z: impedance
R: resistance
X: reactance
CPE: constant phase element
NaOH-NALC: sodium hydroxide-N-acetyl-l-cysteine

## References

[1] World Health Organization. Global tuberculosis report 2014. http://www.who.int/tb/publications/global_report/en/ (2014). Accessed 25 June 2015.

[2] Chan WC, Li LK, Yung PT. Wearable patch for the electrical, durometry and optical skin measurements in tuberculin skin test. In: Biomedical and health informatics (BHI), 2012 IEEE-EMBS international conference on 2012. IEEE.

[3] Parsons LM (2011). Laboratory diagnosis of tuberculosis in resource-poor countries: challenges and opportunities. Clin Microbiol Rev.

[4] He F (2003). A rapid method for determining *Mycobacterium tuberculosis* based on a bulk acoustic wave impedance biosensor. Talanta.

[5] Cui H (2013). An AC electrokinetic impedance immunosensor for rapid detection of tuberculosis. Analyst.

[6] Noordhoek GT (1994). Sensitivity and specificity of PCR for detection of *Mycobacterium tuberculosis*: a blind comparison study among seven laboratories. J Clin Microbiol.

[7] De Beenhouwer H (1995). Rapid detection of rifampicin resistance in sputum and biopsy specimens from tuberculosis patients by PCR and line probe assay. Tuber Lung Dis.

[8] Dalovisio JR (1996). Comparison of the amplified *Mycobacterium tuberculosis* (MTB) direct test, Amplicor MTB PCR, and IS6110-PCR for detection of MTB in respiratory specimens. Clin Infect Dis.

[9] Helb D (2010). Rapid detection of Mycobacterium tuberculosis and rifampin resistance by use of on-demand, near-patient technology. J Clin Microbiol.

[10] Pozzato N (2011). Evaluation of a rapid and inexpensive liquid culture system for the detection of *Mycobacterium avium* subsp. paratuberculosis in bovine faeces. J Microbiol Methods.

[11] van Zyl-Smit RN (2011). Comparison of quantitative techniques including Xpert MTB/RIF to evaluate mycobacterial burden. PLoS ONE.

[12] Harausz E (2015). Comparison of MGIT and Myco/F lytic liquid-based blood culture systems for recovery of *Mycobacterium tuberculosis* from pleural fluid. J Clin Microbiol.

[13] World Health Organization. Frequently asked questions on xpert mtb/rif assay. World Health Organization, Geneva, Switzerland. http://www.who.int/tb/laboratory/xpert_faqs.pdf, 2015.

[14] Association of Public Health Laboratories. National TB Services Survey Report. 2012. https://www.aphl.org/programs/infectious_disease/Documents/NationalTBReport_June2012.pdf.

[15] Wilson ML (2011). Recent advances in the laboratory detection of *Mycobacterium tuberculosis* complex and drug resistance. Clin Infect Dis.

[16] Pai M, Kalantri S, Dheda K (2006). New tools and emerging technologies for the diagnosis of tuberculosis: part II. Active tuberculosis and drug resistance. Expert Rev Mol Diagn.

[17] Pfyffer GE (1997). Comparison of the mycobacteria growth indicator tube (MGIT) with radiometric and solid culture for recovery of acid-fast bacilli. J Clin Microbiol.

[18] Tortoli E (1999). Use of BACTEC MGIT 960 for recovery of mycobacteria from clinical specimens: multicenter study. J Clin Microbiol.

[19] Romano L (2002). Early detection of negative BACTEC MGIT 960 cultures by PCR-reverse cross-blot hybridization assay. J Clin Microbiol.

[20] Aggarwal P (2008). Comparison of the radiometric BACTEC 460 TB culture system and Löwenstein-Jensen medium for the isolation of mycobacteria in cutaneous tuberculosis and their drug susceptibility pattern. Int J Dermatol.

[21] Yuksel P, Saribas S, Bagdatli Y (2011). Comparison of the VersaTrek and BACTEC MGIT 960 systems for the contamination rate, time of detection and recovery of mycobacteria from clinical specimens. Afr J Microbiol Res.

[22] Sharma B (2010). Evaluation of a rapid differentiation test for *Mycobacterium tuberculosis* from other mycobacteria by selective inhibition with p-nitrobenzoic acid using MGIT 960. J Lab Phys.

[23] Angeby KA (2003). Evaluation of the BacT/ALERT 3D system for recovery and drug susceptibility testing of *Mycobacterium tuberculosis*. Clin Microbiol Infect.

[24] Sengupta S, Battigelli DA, Chang HC (2006). A micro-scale multi-frequency reactance measurement technique to detect bacterial growth at low bio-particle concentrations. Lab Chip.

[25] Stewart GN (1899). The charges produced by the growth of bacteria in the molecular concentration and electrical conductivity of culture media. J Exp Med.

[26] Cole KS (1928). Electric impedance of suspensions of spheres. J Gen Physiol.

[27] Rosen D. 6—Dielectric measurements of proteins. In: A laboratory manual of analytical methods of protein chemistry. London: Pergamon; 1966. p. 191–222.

[28] Richards JC (1978). Electronic measurement of bacterial growth. J Phys E.

[29] Ur A, Brown DF (1975). Impedance monitoring of bacterial activity. J Med Microbiol.

[30] Cady P (1975). Rapid automated bacterial identification by impedance measurement.

[31] Felice CJ, Valentinuzzi ME (1999). Medium and interface components in impedance microbiology. IEEE Trans Biomed Eng.

[32] Markx GH, Davey CL (1999). The dielectric properties of biological cells at radiofrequencies: applications in biotechnology. Enzyme Microb Technol.

[33] Muñoz-Berbel X, et al. Impedance-based biosensors for pathogen detection. In: Principles of bacterial detection: biosensors, recognition receptors and microsystems. Berlin: Springer; 2008. p. 341–76.

[34] Basuray S, Chang HC (2007). Induced dipoles and dielectrophoresis of nanocolloids in electrolytes. Phys Rev E.

[35] Lvovich VF. Fundamentals of electrochemical impedance spectroscopy. In: Impedance spectroscopy. New York: Wiley; 2012. p. 1–21.

[36] Puttaswamy S, Sengupta S (2010). Rapid detection of bacterial proliferation in food samples using microchannel impedance measurements at multiple frequencies. Sens Instrum Food Qual Saf.

[37] Puttaswamy S, Lee BD, Sengupta S (2011). Novel electrical method for early detection of viable bacteria in blood cultures. J Clin Microbiol.

[38] Banada PP, Koshy R, Alland D (2013). Detection of *Mycobacterium tuberculosis* in blood by use of the Xpert MTB/RIF assay. J Clin Microbiol.

[39] Connell ND (1995). Chapter 6: Mycobacterium: Isolation, Maintenance, Transformation, and Mutant Selection. Methods Cell Biol.

[40] Gill WP (2009). A replication clock for *Mycobacterium tuberculosis*. Nat Med.

[41] Gordon RE, Smith MM (1953). Rapidly growing, acid fast bacteria I.: species’ descriptions of *Mycobacterium phlei* Lehmann and Neumann and *Mycobacterium smegmatis* (Trevisan) Lehmann and Neumann1. J Bacteriol.

[42] Smith I (2003). *Mycobacterium tuberculosis* pathogenesis and molecular determinants of virulence. Clin Microbiol Rev.

[43] Babady NE, Wengenack NL. Clinical laboratory diagnostics for *Mycobacterium tuberculosis*. INTECH Open Access Publisher; 2012.

[44] Manual of clinical microbiology (2015). Washington.

[45] Murugasu-Oei B, Dick T (2000). Bactericidal activity of nitrofurans against growing and dormant *Mycobacterium bovis* BCG. J Antimicrob Chemother.

[46] Bettencourt P (2010). Application of confocal microscopy for quantification of intracellular mycobacteria in macrophages.

[47] Chan CE (2013). Novel phage display-derived mycolic acid-specific antibodies with potential for tuberculosis diagnosis. J Lipid Res.

[48] Puttaswamy S (2012). Novel electrical method for the rapid determination of minimum inhibitory concentration (MIC) and assay of bactericidal/bacteriostatic activity. J Biosens Bioelectron S.

[49] Hinton PR. Statistics Explained. New York: Routledge, Taylor and Francis Group; 2014.

[50] McClean M (2011). Identification and characterization of breakthrough contaminants associated with the conventional isolation of *Mycobacterium tuberculosis*. J Med Microbiol.

[51] Kubica G (1963). Sputum digestion and decontamination with N-acetyl-l-cysteine—sodium hydroxide for culture of mycobacteria. Am Rev Respir Dis.

[52] Chatterjee M (2013). Effects of different methods of decontamination for successful cultivation of *Mycobacterium tuberculosis*. Indian J Med Res.

[53] Sharma M (2012). Comparison of modified Petroff’s and N-acetyl-l-cysteine-sodium hydroxide methods for sputum decontamination in tertiary care hospital in India. Med J Dr DY Patil Univ.

[54] Carroll KC, Jorgensen JH, Pfaller MA (2015). Manual of clinical microbiology.

[55] Burdz TVN, Wolfe J, Kabani A (2003). Evaluation of sputum decontamination methods for *Mycobacterium tuberculosis* using viable colony counts and flow cytometry. Diagn Microbiol Infect Dis.

[56] Steingart KR (2006). Sputum processing methods to improve the sensitivity of smear microscopy for tuberculosis: a systematic review. Lancet Infect Dis.

[57] Piersimoni C, Scarparo C (2003). Relevance of commercial amplification methods for direct detection of *Mycobacterium tuberculosis* complex in clinical samples. J Clin Microbiol.

